# Modeling of Entangled Polymer Diffusion in Melts and Nanocomposites: A Review

**DOI:** 10.3390/polym11050876

**Published:** 2019-05-14

**Authors:** Argyrios Karatrantos, Russell J. Composto, Karen I. Winey, Martin Kröger, Nigel Clarke

**Affiliations:** 1 Materials Research and Technology, Luxembourg Institute of Science and Technology, 5, Avenue des Hauts-Fourneaux, L-4362 Esch-sur-Alzette, Luxembourg; 2 Department of Materials Science and Engineering, University of Pennsylvania, Philadelphia, PA 19104, USA; composto@seas.upenn.edu (R.J.C.); winey@seas.upenn.edu (K.I.W.); 3 Polymer Physics, Department of Materials, ETH Zurich, Leopold-Ruzicka-Weg 4, CH-8093 Zurich, Switzerland; 4 Department of Physics and Astronomy, University of Sheffield, Sheffield S3 7RH, UK

**Keywords:** entangled polymer diffusion, molecular dynamics, brownian dynamics, Monte Carlo, slip-spring models, mesoscale simulation, atomistic simulation, microscale simulation, experiments

## Abstract

This review concerns modeling studies of the fundamental problem of entangled (reptational) homopolymer diffusion in melts and nanocomposite materials in comparison to experiments. In polymer melts, the developed united atom and multibead spring models predict an exponent of the molecular weight dependence to the polymer diffusion very similar to experiments and the tube reptation model. There are rather unexplored parameters that can influence polymer diffusion such as polymer semiflexibility or polydispersity, leading to a different exponent. Models with soft potentials or slip-springs can estimate accurately the tube model predictions in polymer melts enabling us to reach larger length scales and simulate well entangled polymers. However, in polymer nanocomposites, reptational polymer diffusion is more complicated due to nanoparticle fillers size, loading, geometry and polymer-nanoparticle interactions.

## 1. Introduction

While there are numerous simulation studies investigating polymer structure [1,2,3,4,5,6,7], reinforcement [8,9,10], entanglements [11,12,13,14,15] and their effect on macroscopic properties, such as viscosity [16,17,18], and mechanical properties [19], on the contrary, there are much fewer studies focusing to the simulation of entangled polymer diffusion [20]. The rate of polymer diffusion of entangled polymers is important since it is a critical factor for the quality of polymer blends and nanocomposites. The tube (reptation) model [21,22,23,24] claims that the movement of a long polymer chain is confined to and moves along a tube, which is formed by the entanglements of neighboring chains. Implications of the reptation model have been worked out during the last decades. As an example, the mean square displacement of a Rouse segment $\phi \left(t\right)=\langle {\left[R\left(t\right)-R\left(0\right)\right]}^{2}\rangle$ within a chain with *N* segments, segment length *b*, where R(t) denotes the spatial position of the segment, is: $\phi \left(t\right)/Nb^{2}∼{\left(t/\tau_{R}\right)}^{1/2}$ for $t\leq \tau_{e}$, ∼${\left(t/Z^{2}\tau_{R}\right)}^{1/4}$ for $t\in [\tau_{e},\tau_{R}]$, ∼${\left(t/\tau_{d}\right)}^{1/2}$ for $t\in [\tau_{R},\tau_{d}]$, and ∼$\left(t/\tau_{d}\right)$ for $t\geq \tau_{d}$. Here $\tau_{e}$, $\tau_{R}$, and $\tau_{d}$ are the times at which the segment begins to feel the tube constraint, the Rouse relaxation time, and the disentanglement time, respectively. In particular $\tau_{R}∼N^{2}$, $\tau_{e}≃\tau_{R}/Z^{2}$, $\tau_{d}≃Z\tau_{R}$, where $Z∼N/N_{e}$ denotes the number of entanglements per chain, and $N_{e}$ the entanglement length, which is affected by density and semiflexibility of polymer chain. The self-diffusion coefficient of a reptating polymer chain is $D∼N^{-2}$ according to the tube reptation model, in contrast to the one for a Rouse: $D∼N^{-1}$ or Zimm chain $D∼N^{-\nu }$ involving the Flory exponent ν. Taking into account contour length fluctuations, $\tau_{d}$ has been revised by Doi [21] as $\tau_{d}\to \tau_{d}{\left[1-X/\sqrt{Z}\right]}^{2}$ where *X* is a numerical factor (X>1.47). Corresponding differences between the viscoelastic properties, relaxation modulus, dynamic light scattering of entangled and unentangled polymer melts have been discussed in detail in the literature [21,25].

In polymer melts, the reptation model has been confirmed and tested by both equilibrium [11,26,27] and nonequilibrium simulations [28,29,30,31], and experiments [32,33,34]. The problem of entangled polymer diffusion [35] with obstacles (nanoparticles) has been addressed by a few studies, but in nanocomposites, there are several parameters that can affect the reptational tube, such as nanoparticle type or loading, confinement, polymer–nanoparticle type of interaction [36]. In this article we do not aim to report and discuss studies that investigate the dynamics of polymers under the presence of obstacles or confinement. Neither is the goal to report in detail, experimental efforts [37] nor the details of different molecular simulation techniques for measuring polymer diffusion. Here is a thorough review of theoretical research and methodologies that have been implemented to address and investigate the reptational linear homopolymer diffusion, in melts and nanocomposites, in comparison to experiments. This review is organized to discuss in order from atomistic to mesoscale and theoretical modeling of polymer melts and nanocomposites (including polymers confined between surfaces).

## 2. Experiments

### 2.1. Polymer Melts

Early measurements of the polymer chain self-diffusion coefficient *D*, by forward recoil spectrometry, in highly entangled poly(styrene) (PS) melts, observed a scaling relation $D∼M^{-2}$ [38], later refined to $D∼M^{-2.28}$ [39] where *M* is the molecular weight of a PS molecule. The tracer diffusion coefficients $D^{*}$ of deuterated PS diffusing into a poly(styrene) matrix of molecular weight *P* was measured by means of an ion-beam analysis method [40,41]. For matrices with molecular weight P> 200 kDa, the tracer diffusion coefficients $D^{*}$ values were independent of *P*, which is consistent with the reptation mechanism. As the molecular weight *P* of the matrix is decreased, tracer diffusion coefficient $D^{*}$ increases due to a constraint release mechanism [40] that arises from the diffusion of the surrounding matrix chains. It was found that the constraint release diffusion behaves as $D∼M^{-1}P^{-3}$ [42,43]. The constraint release is negligible in the measurement of $D^{*}$ when the matrix molecular weight *P* is sufficiently large. The temperature dependence of $D^{*}/T$ is reflected basically in the monomeric friction coefficient, and is accurately described by the Vogel equation [44]. In other studies of diffusion in PS melts, Antonietti et al. [45,46,47] also verified the Doi-Edwards chain reptation model [22]. The crossover to Rouse-like behavior for short tracer PS chains is at $M_{e}=18$ kDa for higher molecular weight matrices and $M_{e}=33$ kDa when the tracer and matrix molecular weights coincide [46].

**Figure 1:**
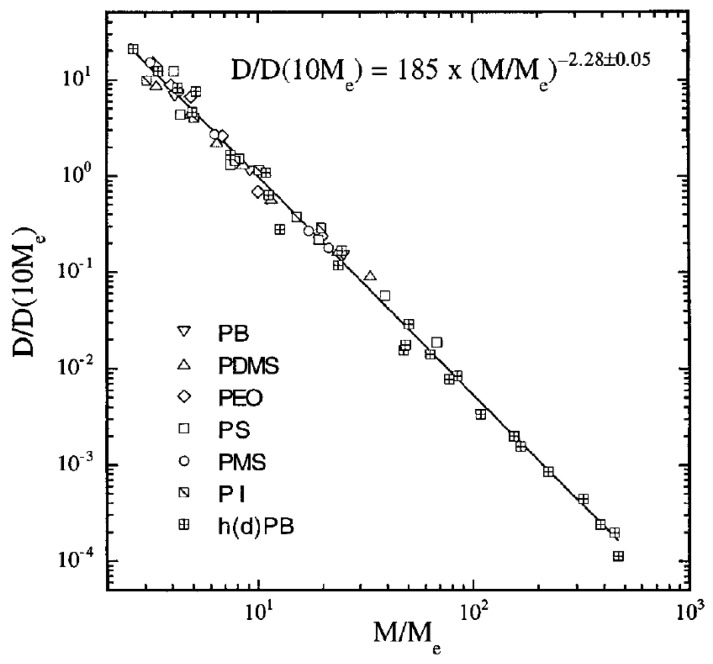
Melt self-diffusion data for hydrogenated (or deuterated) poly(butadiene) samples (hPB and dPB, respectively) adjusted to 175 °C, as a function of molecular weight and data for six other polymers from the literature, with the global power law fit [39,42,43,45,46,48,49,50,51]. Reprinted from [39] with permission from American Institute of Physics (AIP).

In hydrogenated poly(butadiene) (hPB) solutions and melts, it was found that $D∼M^{-2.3\pm 0.1}$ (Figure 1). Although these results contradicted the widespread belief that in melts $D∼M^{-2.0}$ (which was observed for the PS tracer diffusion in highly entangled matrices obtained by Green et al. [42], Green and Kramer [43] and Pearson et al. [52]), it was shown that in fact the literature data for seven different polymers are consistent with $D∼M^{-2.28\pm 0.05}$ scaling relation (Figure 1) [39]. Furthermore, the diffusion coefficient *D* of hPB/alkane solutions was also measured by forward recoil spectrometry. For samples with $M/M_{e}>3$ the data were in accordance to the scaling relation $D\approx M^{-2.4}\phi^{-1.8}$ [53]. The above scaling relations and experiments were included and discussed in more detail in the review by Wang [54].

Other experimental methods that have been applied to measure the diffusion of entangled linear polymers such as poly(butadiene), poly(isoprene) (PI), and poly(dimethylsiloxane) are proton multiple-quantum NMR experiments [55] in combination with time-temperature superposition. Such an experimental method can cover a large range of molecular weight (*M* = 10–200 kDa). In addition, such a method can measure the segmental orientation autocorrelation function which can be compared to tube model theoretical predictions [56]. The disentanglement time was evaluated as $\tau_{d}∼M^{3.3}$ slightly below $\tau_{d}∼M^{3.4}$ as the tube model predicts due to constraint release and contour length fluctuations [57]. The entanglement time that is consistent with rheological results [58] was evaluated by Trutschel et al. [59]. A time scaling exponent of 0.8 of the segmental orientation autocorrelation function in the Rouse regime was calculated, smaller than the theoretical unity Rouse prediction but in good agreement with Monte Carlo simulations, where the measured exponent was 0.83 [60]. It was also found that $\tau_{d}∼M^{3.27}$ which is not in perfect accordance with the tube model predictions. In addition, in that study [59] the self-diffusion coefficients was measured by pulsed-gradient NMR [51,61] and the power law exponent was consistent with earlier studies [49,62]. Moreover, in bidisperse mixtures of 1,4 poly(butadiene), the diffusion coefficient of longer chains decreases by either increasing the chain length of short chains or reducing the weight fraction of short chains in the mixture [62]. These microscopic insights are compared in this review with results from computer simulations and theoretical approaches [59].

### 2.2. Nanocomposites

**Figure 2:**
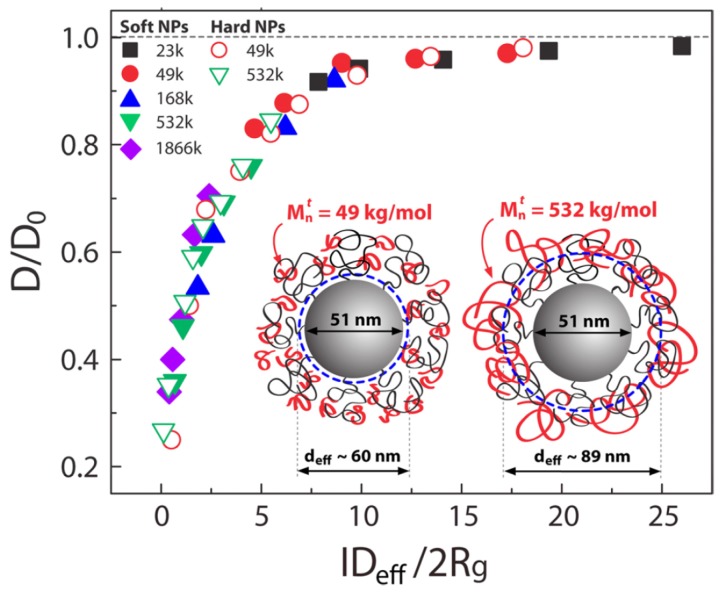
Effect of confinement parameter, defined as the interparticle distance relative to the tracer polymer size, on diffusion. The reduced diffusion coefficient $D/D_{0}$ of deuterated PS (dPS) with molecular weights *M* = 23, 49, 168, 532, and 1866 kDa as a function of the confinement parameter $I\;D_{\mathrm{eff}}/2R_{g}$ yields a master curve. Closed and open symbols represent dPS diffusion in nanocomposites with soft (SiO${}_{2}$–PS87k) and hard (SiO${}_{2}$–I) nanoparticles, respectively. [64]. Reprinted from [64] with permission from the American Chemical Society (ACS).

Detailed information regarding the experimental studies of polymer diffusion in nanocomposites can be found in the reviews by Lin et al. [37] and Schneider et al. [63]. In particular, we refer below the main studies regarding polymer diffusion in nanocomposites containing either spherical (such as nanosilica or gold nanospheres) or anisotropic nanoparticles (such as carbon nanotubes or TiO${}_{2}$ nanorods). The deuterated entangled poly(methyl methacrylate) (PMMA) or PS diffusion was decreased as a function of nanosilica loadings and the normalized diffusion coefficient to the bulk value as a function of the confinement parameter $I\;D_{\mathrm{eff}}/2R_{g}$ collapses into a master curve (Figure 2). Here, $I\;D_{\mathrm{eff}}=I\;D-h_{\mathrm{eff}}$, where ID is the interparticle spacing, $h_{\mathrm{eff}}$ approximates the distance of closest approach for a tracer molecule in the brush and $R_{g}$ is the tracer radius of gyration. Choi et al. [64] studied the diffusion of deuterated PS in the presence of soft nanoparticles (silica nanoparticles with PS tethered chains) and produced the same curve as that of the hard nanosilicas composites [65,66] where $I\;D/2R_{g}$ collapsed the reduced polymer diffusion $D/D_{0}$ data for isothermal measurements which was considered evidence of a mechanism related to entropy. However, this collapse of $D/D_{0}$ breaks down as a function of temperature, and the data does not reduce as well as seen in Figure 2 explaining that mechanism of diffusion is different. In particular, for constant $I\;D/2R_{g}$, *D* is more perturbed from bulk at higher temperatures [67].

For small and mobile NPs in an attractive polymer melt [68], $D/D_{0}$ was more perturbed than in nanocomposites with larger and immobile NPs. Furthermore, the mechanism for slowing the tracer polymer diffusion was related to slowed segmental dynamics (friction) rather than excluded volume or entropy as shown by Tung et al. [67]. In addition, Mu et al. [69] also investigated the diffusion of deuterated PS in PS/single-walled carbon nanotubes (SWCNT) composites and found approximately an order of magnitude decrease with SWCNT below percolation concentration. Beyond percolation the deuterated PS diffusivity increased and then recovered its bulk value. A similar behavior of the deuterated PS diffusion had also been observed for the case of PS–fullerene nanocomposites [70].

In graphene nanocomposites, the diffusion of poly(methyl methacrylate) (PMMA) confined between graphene oxide (GO) sheets was measured by the neutron reflectivity (NR) technique [71,72]. It was shown that the confined PMMA diffusivity was reduced by more than an order of magnitude if the thickness of the film was less than $3R_{g}$ (where $R_{g}$ is the polymer radius of gyration in bulk), whereas the diffusivity of confined poly(styrene) (PS) was reduced by 3 times in comparison to bulk diffusivity, as the film thickness decreased from $8R_{g}$ to $1R_{g}$. That discrepancy was due to PMMA-GO interaction [71]. Furthermore, the self-diffusion of confined poly(butadiene) chains in alumina channels (100 µm long, 20 and 60 nm wide) was investigated [73] by proton pulsed-gradient NMR. A reduction of the diffusivity appeared depending on the pore diameter but not on the molecular weight for short polymers (molecular weight between 2 and 24 kDa). This behavior was rationalized in terms of the monomeric friction coefficient, which suggested a 10-times enhanced friction due to the surface in a single molecular layer [73]. Moreover, by using elastic recoil detection, PS tracer diffusivity along the cylindrical aluminim oxide membranes was measured and an increase in comparison to the bulk value was observed as the confinement increases, in agreement with other studies [74].

## 3. Atomistic Simulations

### 3.1. Polymer Melts

Atomistic simulations of polymer diffusion remain very computationally demanding. However, one of the very few efforts is the long (ms time scale simulations) atomistic molecular dynamics (MD) simulations of monodisperse linear poly(ethylene oxide) (PEO) melts, of molecular weight up to M≈ 20 kDa. The self-diffusion coefficient obtained, by using a united atom force field, was compared quantitatively with experimental data from neutron spin echo and pulse-field gradient NMR [75]. It was shown that the PEO diffusion coefficient (with M>5 kDa) scales as $D∼N^{-2.1}$, the same as for poly(ethylene) (PE) melts [76] for chains up to N=500 monomers in which the dynamic structure factors S(q,t) from simulation data agreed well with neutron scattering experiments [76].

Regarding the PE melt, atomistic MD simulations were performed with a number of monomers from N=78 to N=250. Above N>156, the mean square displacement (MSD) and dynamics structure factor of inner segments showed a change from a Rouse towards a reptation-type behavior [78,79,80,81] with a predicted scaling relation of $D∼N^{-2.4}$ different than the one reported by Hur et al. [76].

**Figure 3:**
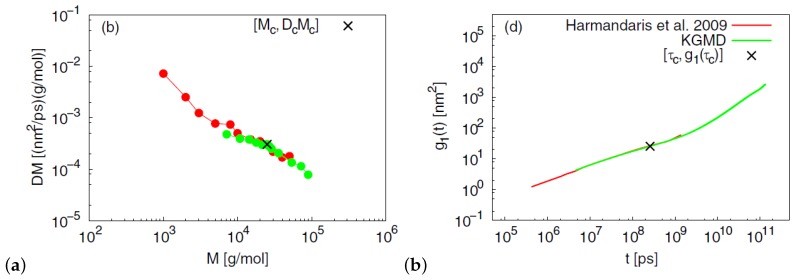
Results of mapping between multiscale MD of PS and Kremer-Grest MD (KGMD). (**a**) Diffusion coefficient times *M* versus molecular weight *M*, (**b**) mean square displacement, $g_{1}\left(t\right)$, of central monomers for 50 kDa PS [77]. Reprinted from [77] with permission from Springer Nature.

A similar effort for PE with larger number of monomers (N=500) was performed by Ramos et al. [82]. The results from simulations regarding the friction coefficient ζ, the zero-shear rate viscosity $\eta_{0}$ and the self-diffusivity were in agreement with experiments [27]. Another MD study of a united atom force field for PE was applied by Takahashi et al. [83]. The scaling laws for representative polymer properties, such as diffusion, viscosity, relaxation time were compared to theoretical predictions [83] as can be seen in Figure 3. Atomistic simulations of cis-1,4-poly(butadiene) (PB) melts, in high molecular length of N=400 monomers, by implementing a united atom force field, were performed for times up to 600 ns [84]. Dynamic properties, such as segmental and terminal relaxation, self-diffusivity and the single-chain dynamic structure factor, S(q,t), at atmospheric pressure in a temperature range of T= 298–430 K were calculated [84]. The data showed that, around N=200 monomers, *D* changed to a reptation-like (where $D∼M^{-2.1}$) behavior at 413 K.

### 3.2. Polymers Confined between Surfaces

In nanocomposites there is a very limited number of polymer diffusion studies by using atomistic simulation (through implementation of a united atom force field) due to the computational demand. In particular, dynamics of confined PE chains between graphite walls was studied by Kritikos et al. [85]. The molecular weight of the monodisperse PE chains was high enough up to the entanglement regime. Three cases of distances between the walls, (2, 3, $4\times R_{g}$), of PE chains were studied. By conducting MD simulations (at temperature T=450 K)—after equilibration by Monte Carlo (MC) algorithms—the relaxation time distribution was a function of distance from the graphite walls. The diffusion of PE chains in the middle layer was not inhibited by the presence of the adsorbed PE layers [85].

## 4. Coarse-Grained Simulations

### 4.1. Molecular Dynamics

#### 4.1.1. Polymer Melts

One of the first studies in microscale simulations by MD was implemented by the simple off-lattice polymer model (freely-jointed tangent hard-sphere chain) in order to perform long time simulations to probe the entangled polymer dynamics. The highest chain lengths that were studied was N=192 segments for a volume fraction between ϕ = 0.3–0.45 [86,87,88]. The inner segments of the mean square displacement (for longest chains) followed the three different scaling regimes of the tube model. In addition, an anomalous diffusive behavior in the atomic MSD of the inner segments was observed as the inner segments cross over into the free diffusion limit [86,87]. Shanbhag [89] studied monodisperse polymer melts via the bond fluctuation model ($N_{e}\approx 30$) and evaluated the diffusion coefficient *D* of chains using the so-called BOOTDIFF method [90], more generally useful for systems with nondiffusive short-time behavior. They obtained diffusion coefficients for N=30, 75, 150 however, they had too few data points to extract a scaling exponent.

**Figure 4:**
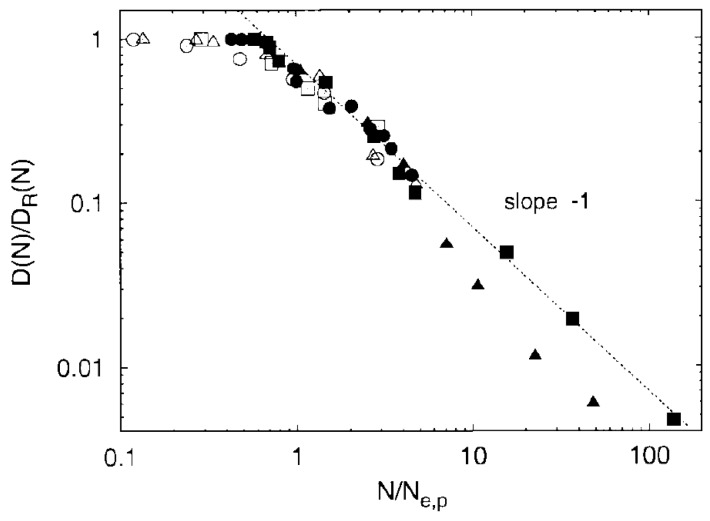
Scaled diffusion coefficient $D\left(N\right)/D_{R}\left(N\right)$ vs. scaled chain length $N/N_{e}$ for poly(styrene) ($M_{e}=14600$, T= 485 K, (filled circles) [46], poly(ethylene) ($M_{e}=870$, T=448 K, filled squares) [52], PEB2 ($M_{e}=992$, T=448 K, filled triangles) [49], bead-spring Kremer-Grest model ($N_{e}=72$, open triangles), the bond-fluctuation model for ϕ=0.5, ($N_{e}=30$, open squares) [91], and tangent hard spheres at ϕ=0.45$N_{e}=29$, open circles) [87]. All data are scaled with $N_{e}$ from the plateau modulus or with 2.25$N_{e}$ from $g_{1}\left(t\right)$. Reprinted from [92] with permission from IOPscience.

In microscale simulations there are numerous studies that use the Kremer-Grest model [26] to investigate polymer dynamics and eventually diffusion. The inclusion of the attractive part of the non bonded Lennard Jones (LJ) potential, between monomers of the chains, had insignificant effect on the chain mobility for a temperature larger than twice the glass transition temperature [94]. However, for lower temperatures the attractive part of the LJ potential reduced polymer diffusion [94] in comparison to that predicted by the Kremer-Grest model (which contains only the repulsive part of the LJ potential) [26]. The search for the reptation power laws [35] as predicted by reptation tube theory was rather difficult [83,94,95,96,97,98,99,100]. In particular it was shown that, by using the Kremer-Grest [26] model, it was difficult to identify any clear power-laws, at least for mildly entangled systems [96] in contrast to the more entangled polymers (N=350) studied by Pütz et al. [92] (Figure 4). The predictions of Doi and Edwards, regarding the four different power laws [101] present in mean square chain of mass displaments, tube diameter, and the time scales could be determined from the intersections of the power law fits.

**Figure 5:**
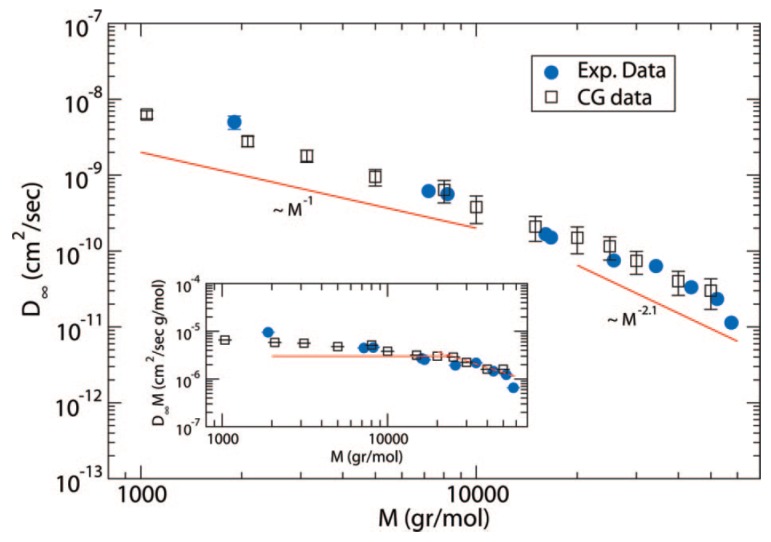
Self-diffusion coefficient of PS melts at T=463 K [93], after correcting for the chain length dependent friction coefficient, $D_{\infty }M$, as a function of the molecular weight. In the inset $D_{\infty }M$ vs *M* is shown at T=463 K [93]. Experimental data are corrected for the different temperature (T=458 K) with the temperature dependence reported in Reference [46]. Reprinted from [93] with permission from ACS.

A hierarchical approach that combined both atomistic and coarse-grained dynamic simulations of entangled PS melts was studied by Harmandaris and Kremer [93] in order to capture dynamical and rheological properties. The time mapping constant was determined by comparing atomistic and coarse-grained simulations for oligomers. The chain self-diffusivity in reptation regime, after correcting for the chain end free volume, were predicted in comparison to experimental data [93] (Figure 5). In another coarse-graining effort, a mesoscale PS model was developed using the iterative Boltzmann inversion (IBI) approach [102] in order to predict the polymer dynamics of long chains. The potential was optimized, according to IBI, until the radial distribution function generated from the mesoscale model was consistent with that produced by the atomistic simulation of oligomers. Mean-squared displacements measurements captured the crossover dynamics from the Rouse to reptation behavior. In addition, the entanglement length of that PS mesoscale model was around 85 monomers at T=450 K in agreement with that of the bead-spring Kremer–Grest model [103].

Furthermore, CG models of PE were developed by the IBI approach with three to six methyl groups per CG bead. It was discussed that pressure corrections were required after the IBI for the generated CG potentials to match the pressure of atomistic melt and transfer to the CG potentials for modeling different temperatures. However, mean-square displacements (MSDs) and stress autocorrelation functions G(t) for PE melts were independent of the use of pressure-corrected potentials by the IBI. The time rescaling factor to match CG and atomistic models was the same for pressure- and non-pressure-corrected CG models, but depended on temperature [104]. In another coarse graining effort of polymer melts, the time and length between CG and atomistic models, using a generic mapping scheme based on power laws, were estimated by Takahashi et al. [77]. That scheme revealed the characteristic length and time between the different scales to link the atomistic PE model and the bead-spring Kremer–Grest (KG) model. That mapping procedure between the PS CG model and the KG model enabled measurement of polymer dynamics up to the subsecond time scale [77]. Furthermore, a coarse-grained poly(vinyl alcohol) (PVA) model with a triple-well bending potential has been developed to predict polymer dynamics, structure and crystallization during cooling [105]. However, it is an open question if such developed CG models are applicable at other temperatures. An interesting method to develop a transferrable coarse grained potential is the energy renormalization method, that has been implemented for PS and PB melts [106,107].

#### Polymer Polydispersity

Most computer simulation studies of entangled polymers had been performed on monodisperse samples. However, polymer synthesis always results in a distribution of molecular weights with PDI = $M_{w}/M_{n}\approx 1.02$–1.04. The effects of polydispersity on entangled PE dynamics melts were studied recently, using a coarse-grained model by Peters et al. [104]. Entangled PE melts with chain lengths of average $M_{w}=36$ kDa with PDI = 1.0–1.16, were studied for long scale times (600–800 ms) using MD simulations. It was found that polydispersity in that range did not alter the entanglement time or tube diameter [108]. There was a negligible difference in the PE dynamics for the distributions PDI =1.0 and PDI =1.02, however, the shortest chains diffused faster than the longest ones due to a constraint release mechanism [108]. It is worth to note an atomistic simulation effort (using a united atom force field) of polydisperse linear poly(ethylene) melts with PDI=1.09, showed a Rouse-scaling of $D∼N^{-1}$ for chains above N=60 and a signature for the onset of a reptation regime at N=150 [109]. In addition, a detailed study of bidisperse polymer melts had been performed by Wang and Larson [80]. The diffusion coefficient of long chains can be given from two different relations, Equations (1) and (2).

(1) $$\begin{array}{ccc} D_{L} & = & D_{L}^{\mathrm{rep}}\left(1+\alpha_{\mathrm{cr}}N_{e}^{2}N_{L}N_{S}^{-3}\right)=D_{L}^{\mathrm{rep}}\left(1+\alpha_{\mathrm{cr}}r_{\mathrm{GS}}\right), \end{array}$$

(2) $$\begin{array}{ccc} D_{L} & = & D_{L}^{\mathrm{rep}}\left(1+KN_{e}^{3/2}N_{L}N_{S}^{-5/2}\right) \end{array}$$

**Figure 6:**
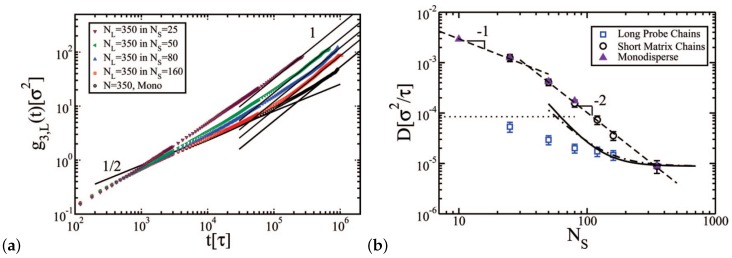
(**a**) Mean square displacement (MSD) of center of mass $g_{3}\left(t\right)$ of the long probe chains with N=350 monomers in binary blends with various short matrix chains $N_{s}$ [80]. (**b**) Diffusion coefficients of the long probe chains with length N=350 and the short matrix chains, as a function of the matrix chain length of the short chains, $N_{s}$, in binary blends with fixed long chain segment fractions $n_{L}=15\%$. The triangles are simulation results obtained from monodisperse melts with fixed chain segment number density ρ=0.85 (Lennard-Jones units). The thick solid and dotted-dashed lines are the predictions of Equations (1) and (2), respectively, using the *z* and *K* values from the experimental work of Green and Kramer [43]. The dotted horizontal line shows the Rouse diffusivity of the probe chains, attained in the matrix offering only frictional, and not topological, resistance to the long chain motion. Reprinted from [80] with permission from ACS.

The mean square displacement and the linear diffusive regime of long polymer chains in the bidisperse melt are depicted in Figure 6a; diffusion coefficients are reported in Figure 6b. The ratio of polymer diffusion coefficient of long chains in a bidisperse sample to the diffusion coefficient of long chains in a monodisperse sample can be predicted by mesoscale simulations [110] in agreement with experiments by Wang et al. [62] for poly(butadiene) (PB) chains for volume fractions of long chains ϕ=20% and 10%.

#### Polymer Semiflexibility

**Figure 7:**
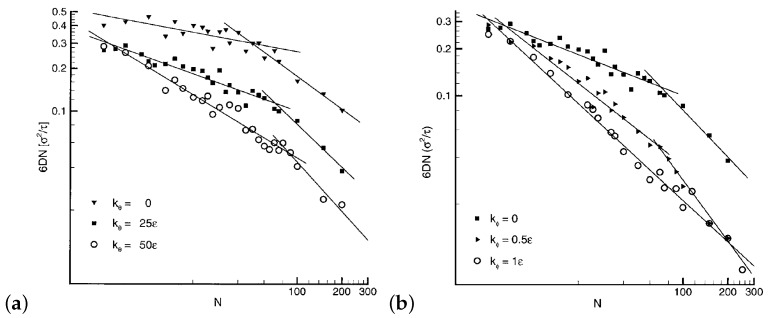
(**a**) The dependence of 6DN on *N* for different bending rigidities in the absence of torsion stiffness, $k_{\phi }=0\epsilon$. (**b**) The dependence of 6DN on *N* for different torsion rigidities, at a constant bending strength $k_{\theta }=25\epsilon$. Reprinted from [111] with permission from ACS.

It has been shown by simulations that the semiflexibility of chains (through the insertion of a bending potential along the backbone of the polymer model) reduces polymer diffusion [111,112], since it increases the Kuhn length [113] and packing length which unavoidably decrease the entanglement length [11,114]. The polymer self-diffusivity decreases when the bending and torsion potentials increase (thus the semiflexibility increases) as can be seen in Figure 7. In addition, semiflexibility moves the transition from Rouse to reptation regimes [81,115] to longer chains. In the reptation regime the power law scaling exponent, of diffusion coefficient, becomes −2.2 for the cases $k_{\theta }=25\epsilon$, $k_{\phi }=0\epsilon$ or $k_{\phi }=0.5\epsilon$, (whereas the exponent becomes −2.1 for $k_{\theta }=50\epsilon$, $k_{\phi }=0\epsilon$) [111] in agreement with experimental [39,53], theoretical [116] and simulation predictions [117].

In a recent study, the dynamic scaling power laws of long semiflexible polymers are investigated [98]. The relaxation times in the Rouse and reptation regime is extracted by the mean square displacements, $g_{1}\left(t\right)$, $g_{2}\left(t\right)$, $g_{3}\left(t\right)$ in quantitative agreement with theoretical predictions. The MSD data for long semiflexible chains N=2000 follows exactly the reptation theory [98]. As can be seen from Figure 7, the exponent of the diffusion molecular weight dependence, in the reptation regime, is influenced by the semiflexibility of the chains and reaches the value of −2.2 for certain stiffness [111].

A higher temperature leads to an increase of the polymer chain diffusion coefficient, independent of chain stiffness [112]. However, the chain diffusion coefficient dependence on chain length is influenced strongly by chain stiffness and temperature [112]. By increasing the stiffness, or decreasing the temperature, the exponent of the chain length dependence decreases approaching the −2 value for unentangled and mildly entangled chains [112]. Furthermore, by adding strongly attractive nanoparticles in a mildly entangled matrix, it is shown that a semiflexible polymer may diffuse faster than a flexible one, following a mechanism of nanoparticle’s attraction weakening with chain semiflexibity [118].

#### 4.1.2. Nanocomposites

**Figure 8:**
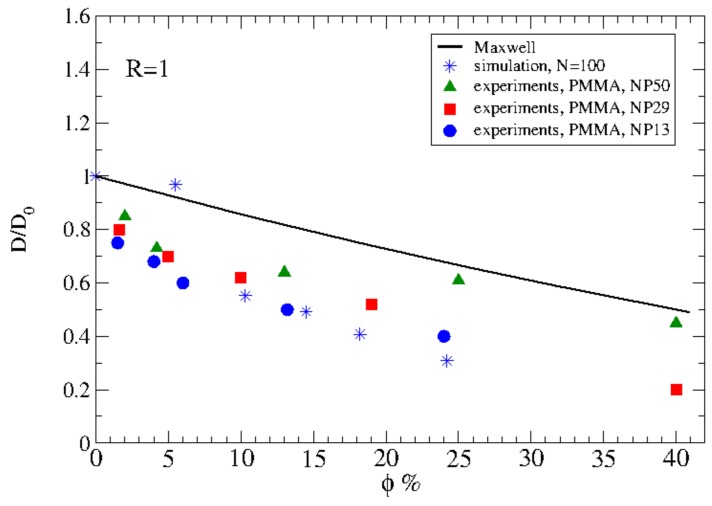
Dependence of diffusivity (normalized to its bulk value) of mildly entangled polymers (N=100) for different nanoparticle loading:(i) Maxwell prediction (black line) (ii) simulation data N=100 (stars), (iii) PMMA tracer diffusion: diameter $d_{\mathrm{NP}}=13$ nm (circles), (iv) $d_{\mathrm{NP}}=29$ nm (squares), (v) $d_{\mathrm{NP}}=50$ nm (triangles). Reprinted from [119] with permission from ACS.

Mildly entangled polymer diffusion in nanocomposites containing spherical nanoparticles [119,120] or nanorods [121] was studied by MD simulations recently. It was shown that the polymer diffusivity decreases due to nanoparticle loading, as can be seen in Figure 8, due to an increase of the interfacial area created by the nanoparticles [119,120]. That recent work was the only one that has been performed for mildly entangled polymers diffusion in nanocomposites by molecular dynamics, while the work of Desai et al. [122] showed a similar behavior with nanoparticle loading for polymers with number of monomers just below the entanglement length.

A few studies have been implemented regarding diffusion in polymers confined between surfaces. In particular, long polymer chains disentangle under the influence of the confinement [123]. It is shown that there is a minimum in the relaxation time (calculated by measuring the correlation function of the end-to-end vectors, [124]) of long chains when decreasing the film thickness, which partially originates by the disentanglement of chains due to the confinement [123,124]. In a recent study of polymers confined in cylindrical geometry the number of entanglements decreases with cylindrical confinement in comparison to thin films [123]. The theoretical prediction of polymer diffusivity in a cylindrical geometry is in excellent agreement with the coarse grained simulations [125,126,127] however, it overpredicts the experimental measurements of PS in alumina oxide membranes [126]. As the diameter of the cylindrical confinement decreases, the chain diffusion coefficient increases (in comparison to bulk) due to chain disentanglement, however, for small pores (<5 times of the monomer segment), diffusivity decreases due to chain segregation [127]. In another recent work with a diamond network confinement it is also shown that polymer diffusivity increases in comparison to the bulk value due to the disentanglement that occurs from the nano-confinement [128], similar to the conclusion by previous studies [123,124].

### 4.2. Monte Carlo

#### 4.2.1. Polymer Melts

In addition to the molecular dynamics studies of polymer diffusion there are quite a few studies implemented by Monte Carlo simulations. Rubinstein [129] proposed a “discretized” reptation model. The tube was modeled by a one-dimensional lattice and the polymer was modeled by a cluster of walkers, called reptons. Each repton represented a part of the chain and was allowed to hop between neighboring sites, but the cluster always remained connected. The repton model predicted that diffusion coefficient scaled as $D∼M^{-2}+O\left(M^{-3}\right)$ and viscosity scaled as $\eta_{0}∼M^{3.4}$ [129]. The dynamics of a polymer in a network of entanglements was also studied by Deutsch and Madden [130]. The diffusion coefficient *D* for chains up to 100 links, scaled as $L^{-2.50\pm 0.04}$, where *L* was the chain length. This result was different than the scaling predictions based on the tube model in which the power law dependence follows the $D∼L^{-2}$ scaling. In that particular work [130], they calculated a three-dimensional diffusion coefficient (previous work used an one-dimensional coefficient [129]). It was noted that the tube length fluctuations in the reptation model were necessary to make a direct comparison to experimental data [130] whereas were neglibile for the one-dimensional coefficient.

The crossover region from the unentangled dynamics to reptation dynamics [131,132] that had been observed from MD, was also verified by using the bond fluctuation model [133,134] in MC simulations. In particular, a study of the cubic lattice model by MC simulations (that contained both excluded volume interactions and entanglements) for polymer volume fractions in the range 0.025<ϕ<0.50 and chain lengths in the range 20<N<200, the crossover from Rouse behavior to reptation [135] was also observed. MC simulations for monodisperse linear polymer chains in dense melts with number of monomers to N=512 were implemented and standard mean-square displacements of inner monomers and of the chain’s center of mass were calculated [133,134]. The analysis revealed that the crossover from unentangled to entangled dynamics was very lengthy [136]. Even for the largest chain length of N=512, no clear evidence for reptational diffusion [132] was shown. The scaling relation of $D\propto N^{-2.1}$ which was very close to the experimental prediction, was predicted over a range of melt densities [137]. A scaling relation of $D\propto N^{-2.2}$ was calculated for PE melts [138] and the start of crossover to reptation regime was observed for N>85 monomers. A different lattice Monte Carlo algorithm for bulk polymer melts had been developed by Shaffer [139]. The topology of the polymer chain could be altered without pertubing the static properties (end-to-end distance, radius of gyration). Such an algorithm can predict the tube model scaling for the polymer diffusion coefficient for a non chain crossing condition [139].

#### 4.2.2. Nanocomposites

**Figure 9:**
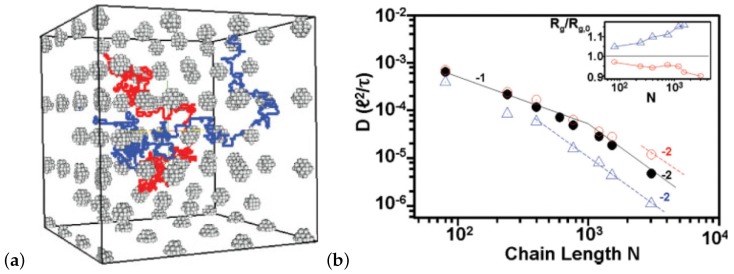
(**a**) Model picture of a nanocomposite system, with nanoparticles of radius R=2σ (where σ is the length of a chain segment) in a body-centered cubic configuration with periodic boundary conditions. (**b**) Dependence of *D* on chain length *N* in the presence of different particles, at different nanoparticle volume fractions ϕ: ϕ=30% of small particles of radius R=2σ (upper triangles), ϕ=40% of large particles with radius R=22σ (open circles), and pure melt (filled circles). Reprinted from [145] with permission from Wiley.

Early MC and theoretical studies [140,141] had shown that polymer diffusion through heterogeneous media was decreased due to the entropy loss that was created by fixed obstacles. The spacing between the neighboring nanoparticles was the main parameter for slowing down of polymer diffusion in nanocomposites. For random walk polymers of length *N* which diffused in randomly distributed obstacles, the chains diffused slower than the reptation model predictions [132] following a scaling relation: $D\approx N^{-2.4}$ [142]. Using the bond fluctuation model, of a single polymer chain in regularly distributed obstacles confined in two dimensions (2D), the mean square displacement of a center monomer $\phi_{M/2}\left(t\right)$ exhibited four dynamics regimes from shortest to longest time (diffusion) regimes [143]. In particular, $\phi_{M/2}\left(t\right)∼t^{\beta }$ where β∼ 3/5, 3/8, 3/4, 1 [143]. The second and third regimes were described by segmental diffusion in the self avoiding tube [143]. In a more recent study on lattice MC simulations of long polymers in nanocomposites [144,145] a decrease in polymer chain diffusion was observed when the nanoparticles (of radius smaller than $R_{g}$) were fixed in a BCC fashion in the polymer matrix, due to confinement and enthalpic effects, as can be seen in Figure 9.

A phenomenological trap model [69,70] was developed to calculate the PS diffusion in SWCNT (or fullerene) nanocomposites [6,146]. The authors assumed that there exists a volume within which the SWCNTs exert an influence on the polymers, which might be considered to be of the order of up to a radius of gyration away from the nanotube surface [69,70]. Diffusion occurs according to the following rules: a jump between two adjacent sites on a cubic lattice occurs with equal probability if the two sites are both inside or both outside the volume influenced by the nanotube, but with a reduced probability if the two sites are in different regions. When the nanotubes are isolated (dilute SWCNT loading) then any polymers within the nanotube influenced regions effectively become trapped with a residence time that depends on the probability of crossing between regions, and long-time diffusion is reduced. Once these regions start to interconnect though, polymers can diffuse long distances regardless of which region they are in so that the influence of the reduced probability of crossing diminishes. As a consequence, the diffusion coefficient starts to increase with concentration above this percolation threshold. The model does not explicitly account for the physical obstacles presented by the SWCNTs, an affect which at the concentrations of interest, up to 5% by volume is expected to be much smaller than the observed effect. The model also does not account for any polymer specific motions, such as reptation. Each diffusing polymer is treated as a single point particle. Despite the simplicity of the model, remarkably it predicts that the lowest possible reduction in the diffusion coefficient, *D* (in the nanocomposite), compared to $D_{0}$ (in the bulk PS), the diffusion coefficient in the absence of SWCNTs, corresponds to $D/D_{0}=0.6$, a value that was subsequently found to be in excellent agreement with the more refined temperature controlled experiments carried out by Tung et al. [147]. This quantitative agreement suggests that the model captures the correct long time diffusion behavior. It does not, however, provide a molecular explanation for what causes the effective barrier for diffusion between the two regions.

#### 4.2.3. Polymers Confined between Surfaces

The effect of bidispersity on the dynamics of polymer films capped between two neutral walls was studied by Li et al. [148] for various compositions. More specifically, they investigated a coarse-grained bond-fluctuation model via Monte Carlo. Their results rendered the characteristic entanglement length to be an important parameter that helped to interpret the effect of bidispersity on the dynamics of their model polymer films. For chains short compared with the characteristic entanglement length, and independent on the film composition, the diffusivity of the short chains was limited, caused by an average number of near-neighboring particles that increased with the decrease of the film thickness. On the contrary, the dynamics of longer chains was determined by the film’s composition. They found that with a lower weight fraction of long chains, the self-diffusion coefficient of long chains decreased slowly with the decrease of the film thickness, which was similar to that of short chains.

## 5. Mesoscale Simulation Methods

### 5.1. Dissipative Particle Dynamics

Dissipative particle dynamics (DPD) is a mesoscopic simulation technique used to predict mainly the morphology in polymer melts, blends, composites. The main feature of DPD simulations is the nature of the conservative force (non bonded force that acts between two particles). In this case the conservative force is not a Lennard–Jones force [149,150] (as in MD) but rather a harmonic force which decreases linearly if the particle distance [151,152] increases. However, this enables chain crossing and as a consequence polymers behave as phantom chains [153,154] thus the reptation scaling laws cannot be predicted [155]. However, there are a few DPD polymer melt simulation efforts to prevent unphysical polymer chain crossing, for instance by incorporating an additional repulsion based on the distance of closest approach between two bonds [156,157,158,159,160,161,162] following the philosophy by Kumar and Larson [163] and Pan and Manke [164] in order to predict the reptation diffusion in long chains. In particular, a segmental repulsion force is introduced, to reduce the frequency of artificial chain segment crossings, that depends on the distance between the midpoints of the segments, rather than the minimum distance between segments. The scaling power law exponent for the center of mass diffusion coefficient, for number of monomers above the entanglement length, is close to the theoretical value of tube model of −2, but the scaling power law exponent for viscosity is close to +2, which is smaller than the experimental value of +3.4 [164]. Without such segmental repulsion forces, chain segment crossings can occur freely [155], thus the scaling of both diffusion coefficient and viscosity follows the Rouse behavior even for high chain lengths [164].

**Figure 10:**
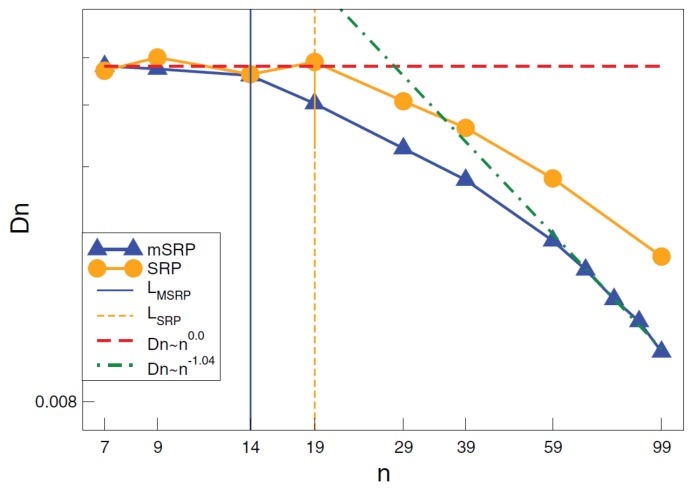
Diffusion as a function of chain length for mSRP and SRP. Rouse (dash) and reptation (dash-dot) scaling limits intersect at approximately N=28 for mSRP. The initial deviation from Rouse diffusion scaling for mSRP and SRP occurs at N=14 (solid) and N=19 (small dash), respectively. Reprinted from [161] with permission from ACS.

An alternative approach that was implemented is the modified segmental repulsive potential (mSRP), whose parameters were determined from topological, structural, and thermodynamic properties. The mSRP potential was able to capture entanglements and the reptational scaling law for diffusion, as shown in Figure 10, in a dense polymer melt [161].

Another approach to avoid unphysical bond crossings is either by introducing the “Twentanglement” algorithm [168,169] or by adding a rigid core around monomers [170] or by using adaptive time stepping [171]. In particular, the idea to create entanglements between coarse grained polymer chains, in the “Twentanglement” algorithm, is to view the bonds as slippery elastic bands with finite extensibility and prevents chains from crossing each other [168]. Following such methodology a chain with N=120 has a non-Rouse behavior [168] but its diffusion is higher than the experimental value of Pearson [52].

**Figure 11:**
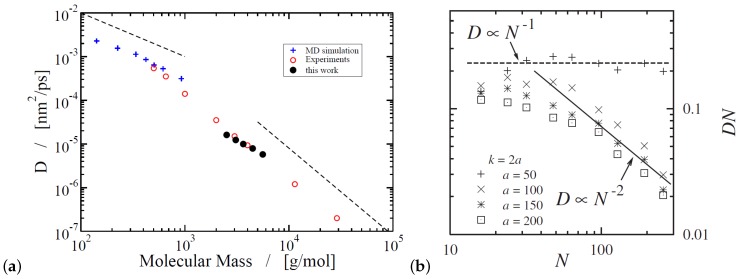
(**a**) Self-diffusion coefficients of linear polymers as a function of molecular mass. The black dots are the results of Fan and Liu [165], the filled circles represent experimental data from Pearson et al. [52], and the plus symbols show molecular dynamics simulation results obtained by Mondello and Grest [166]. The dashed lines illustrate the slopes expected for the Rouse regime (left, $D∼M^{-1}$) and the reptation regime (right, $D∼M^{-2}$). Reprinted from [165] with permission from IOPscience. (**b**) Proper scaling limits are reached for the diffusion coefficient *D*, by DPD simulations (α is the force bond constant). Reprinted from [167] with permission from the American Physical Society (APS).

Moreover, a highly entangled coarsed grained model for PE, containing N=1000 monomers, is developed that is able to form entanglements with neighboring chains and capture the diffusive linear regime at long time scales [172]. In that particular study, the entanglements are implemented as stochastic events, in which the probability of creating an entanglement, in a simulation time step, between a specific pair of coarse-grained chains depends on a weight function (quadratic function) depending the center of mass distance between these two chains [172]. These entanglements can disappear when the chains unknot, move apart or slip off [172]. This is different from the slip-spring model in which creation and annihilation of entanglements depend on the number of monomers at the chain ends [172,173,174]. Following such an approach the polymer diffusion coefficient, which is depicted in Figure 11a, presents a good agreement with the experimental data by Pearson et al. [52] but does not fit exact the power law exponent of −2, indicating that the simulated polymer chains are between Rouse and reptation regimes. Following a different approach, with a geometrical criterion for the entanglements, reptational dynamics at “high” chain length can be obtained [167] (Figure 11b). With such a geometrical criterion, no new length scales or forces need to be added [167] and entanglements are predicted in both melts and nanocomposites [175].

### 5.2. Slip-Springs

Another approach to simulate entangled polymer dynamics was based on the slip-link model (a number of discrete points confine the lateral motion of the polymer chain, while the chain is able to slide through them) which was first developed by Hua and Schieber [176]. This was a combination of the reptation model [21,22] and network model [177]. Based on the framework of the Hua and Schieber model, other different models [56,178,179,180,181] were proposed having different rules for the time evolution of the state variables, and these models could reproduce even quantitatively the linear viscoelastic data of monodisperse linear polymer melts. For instance, the single chain slip-link model by Likthtman [56] was based on the Rubinstein’s and Panyukov’s model [182] for polymer networks. Likthtman’s model contained contour length fluctuations, constraint release, fast Rouse and longitudinal modes, thus it was able to predict chain reptation. Based on that model, Brownian simulations were performed and compared with rheological (linear relaxation, storage and loss moduli, viscosity) and diffusion experiments. The model predictions of those properties were in agreement with experiments measurements for PS, PI, PB.

**Figure 12:**
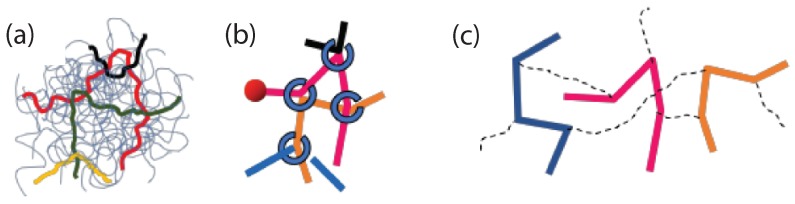
Schematics of slip-spring models. (**a**) Entangled chains colored in an entangled polymer melt, (**b**) corresponding slip-spring primitive chain network (PCN) model [178], and (**c**) a group of chains considered in the Doi and Takimoto (DT) model [179]. Reproduced from [183] with permission from Polymers.

A comparison among different slip-link model simulations to account the constraint release, for bidisperse linear polymer melts, had been implemented by Masubuchi et al. [184] (Figure 12). It was found that the multichain DT model by Doi and Takimoto [179] and the multichain slip link model (primitive chain network (PCN)) by Masubuchi [178] could account for constraint release successfully and compared better to experimental modulus data, for poly(styrene) and poly(butadiene), than the model by Nair and Schieber [180]. Recently, a fast slip link model was implemented for bidisperse linear polymer melts by Shanbhag [185]. Viscosities and dynamic moduli predictions of that fast slip link model [185] were in very good agreement with experimental measurements on 1,4 poly(butadiene)s. The computational time depended on both number of slip-links, $\bar{Z}=M/M_{e}$ (where $M_{e}$ is the entanglement molecular weight), and number of chains, *n*, and scaled as $O(n{\bar{Z}}^{3})$. A numerical study of the slip-link model had been implemented by Biondo et al. [186]. This was used to explore both linear and non-linear rheological properties and furthermore was applied to nanocomposites (containing bare fillers) [186], which could be an alternative to studies implemented by coarse-grained simulations.

An interesting approach in DPD method [191] is to incorporate the topological constraints and, thus uncrossability of polymer chains, by creating temporary cross-links, the so-called slip-spring (variation of the slip link model) following the philosophy of Likhtman [56]. According to this, the lateral motion of the polymer chain is restricted by links connected (by virtual springs) [192] to links on other polymer chains [56,193]. These slip-springs can be created and destroyed at chain ends [56]. Following such a methodology, the polymer chains mean square displacements, diffusivities and longest relaxation time are in agreement with the results of reptation tube theory. By implementing that method, the simulation is 500 times faster than the conventional generic Kremer-Grest model [191] (where unphysical bond crossings are prevented by excluded-volume interactions) which has been used extensively in modeling polymer melts, solutions and nanocomposites by molecular dynamics. That approach of slip-spring model in DPD simulations has also been applied in bidisperse melts in comparison to the conventional KG models. A good agreement in the mean squared displacement measurements is found [110].

The effectiveness of stochastic single-chain models in describing the dynamics of entangled polymers was tested by comparing the slip-spring model to the Kremer-Grest model which was solved using stochastic MD simulations [194]. For a particular chain length in the slip-spring model, the parameters that best reproduced the mean-square displacement were determined by fitting to MD data. Such procedure involved a comparison of the time dependent stress relaxation moduli obtained by both models for a range of chain lengths. The poor agreement of the mean-square monomer displacements at short times observed was corrected by using generalized Langevin equations for the dynamics and that resulted in significantly improved agreement [194]. After identifying a limitation of the original slip-spring model in describing the static structure of the polymer chain, the model was modified so that it agreed with MD results, by introducing a pairwise repulsive potential between the monomers in the chains [194]. The single-chain slip-spring model had been extended by Khaliullin and Schieber [195] to incorporate constraint release in a mean-field way, including polymer fluctuations. The original constraint release concept by de Gennes assumed tube reorganization by Rouse motion of the tube, whose characteristic rate is proportional to the fraction of chain ends, and further determined by reptation of surrounding chains. The inclusion of the additional physics of constraint release [21,196,197] improved the linear viscoelastic predictions of the model, both for monodisperse polymers and bidisperse polymer blends [180,198]. An alternative tube reorganization mechanism, tube dilation, was proposed by Marrucci [199]. Within this concept, reptation of surrounding chains led to a widening of the tube. Constraint release played an important role in the self-consistent determination of the dilated tube diameter. The molecular picture of partial dilation based on this self consistency seemed valid for linear and star chains [200].

**Figure 13:**
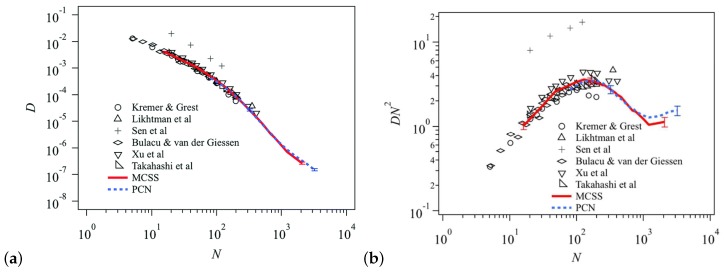
(**a**) Center-of-mass diffusion coefficient and (**b**) its normalized value with respect to the classical reptation behavior (bottom) plotted against the number of beads per chain, *N*. Red solid and blue dotted curves are the results of multi-chain slip-spring (MCSS) and primitive chain network (PCN). The MCSS and PCN data are rescaled by the scale-conversion factors. KG results obtained from the literature [26,96,111,187,188,189] are shown by symbols. Error bar shows the standard deviation for 8 independent simulation runs. Reproduced from [190] with permission from the Royal Society of Chemistry.

A multi-chain extension of the single-chain slip-spring model was introduced [201] that relied on a many-chain representation and introduced the topological effects that arised from the non-crossability of molecules through effective fluctuating interactions, mediated by slip-springs, between neighboring pairs of macromolecules. The total number of slip-springs was not preserved but, instead, it was controlled through a chemical potential that determined the average molecular weight between entanglements. Ramirez-Hernandez et al. [202] extended this model in a way that correctly incorporated the effects of the fluctuating environment in which polymer segments were immersed. The model was used to obtain the equation of state associated with the slip-springs, as well as linear and nonlinear rheology of entangled polymer melts. In another study of the multi-chain slip-spring model [192] the crossover regime between Rouse and entangled dynamics for the polymer chains was observed [192]. In order to test if compatibility between the different properties had been achieved; diffusion, relaxation modulus, entanglement properties were calculated for the multi-chain slip-spring model (primitive chain network model) [178,183,184,190,203] in comparison to the standard Kremer-Grest bead-spring model [26]. In particular, regarding the diffusion, three models were compatible by scaling the units of length, time and number of beads in the polymer chain (Figure 13). However, significant discrepancies were observed for the inter cross-correlations in the relaxation modulus [190].

**Figure 14:**
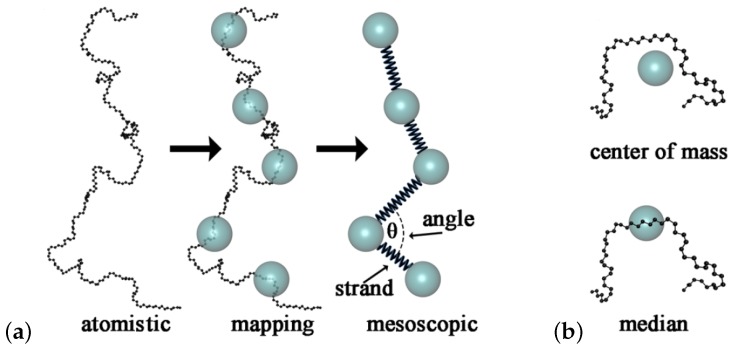
From the atomistic to the mesoscopic representation. (**a**) Mapping of a real polymer chain with 260 carbon atoms onto a sequence of five coarse-grained beads (N=52). (**b**) Two approaches for mapping atomistic polymer segments onto beads. A bead can be assigned either to the center of mass of a segment or to the coordinates of the central atom(s) of a segment. Reprinted from [204] with permission from ACS.

**Figure 15:**
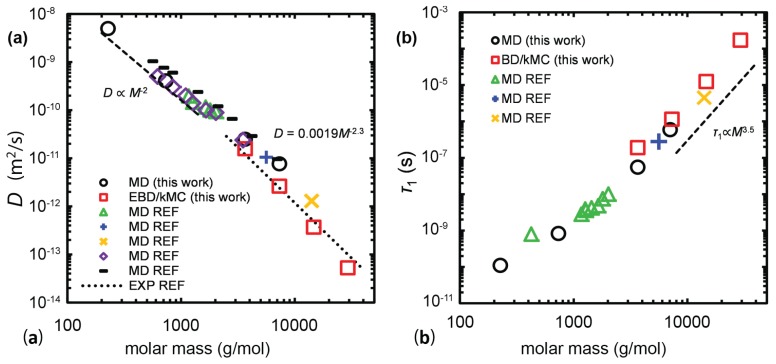
(**a**) Dependence of the self-diffusion coefficient *D* and the longest relaxation time $\tau_{1}$ (obtained from the autocorrelation function of the end-to-end vector) on molar mass, from this work (MD, circles; BD/kMC, red squares) [204], as well as (**b**) from various simulation works in the literature (green triangles [109], blue crosses [206], orange X-markers [207], black dashes [76], purple diamonds [27]). The dotted line is a fit on the experimental data performed by Lodge performed by Lodge [39] while the dashed lines are guides to the eye. Reprinted from [204] with permission from ACS.

Recently, a multiscale simulation strategy that linked detailed MD simulations to slip-springs based on Brownian dynamics/kinetic Monte Carlo (BD/kMC) simulations of long PE chains (N=260 to N=2080) melts was implemented by Sgouros et al. [204] (Figure 14 and Figure 15) and Megariotis et al. [193]. Such (BD/kMC) methodology was based on a Helmholtz energy function that incorporated bonded, slip-spring, and nonbonded interaction terms [205]. The interesting characteristic of such development was that the nonbonded interactions in the absence of slip-springs were derived from an equation of state that was thermodynamically consistent to that obtained by detailed MD and experimental measurements.

## 6. Theoretical Modeling

### 6.1. Polymer Melts

The diffusion of a Gaussian chain in a fixed array of obstacles using the projection operator formalism by Loring [208] was investigated [209]. It was shown that the monomer friction coefficient used in the center of mass mean square displacement could be rewritten exactly in terms of the time correlation function of the total force on the chain. When the decay profile of the force correlation function had an exponential form, and its dependence on the density of obstacles written in an approximate form, the dynamics of the center of mass was found to be subdiffusive at intermediate times and diffusive at long times. Moreover, the diffusion coefficient *D* of the chain at long time scales and at high concentrations of small obstacles was found to vary with monomer number as $D∼N^{-2}$, in qualitative agreement with the predictions of the reptation model [209]. It is worth noting that the self-diffusion coefficient of monodisperse linear polymer melts using an analytical theory of stress relaxation which included contour-length fluctuations predicted a scaling $D∼N^{-2.5}$ relation [116]. This scaling was in qualitative agreement with the polymer mode-coupling theory which predicted scaling relations $\eta ∼N^{-y}$, $D∼N^{-x}$ with exponent values y≥3 and x≥2 for linear polymer melts [210,211,212]. The polymer-mode-coupling (PMC) non reptation theory of Schweizer was able to tune the different exponents of the diffusion coefficient in the crossover to high molecular weight polymers [210,211,212,213].

### 6.2. Nanocomposites

**Figure 16:**
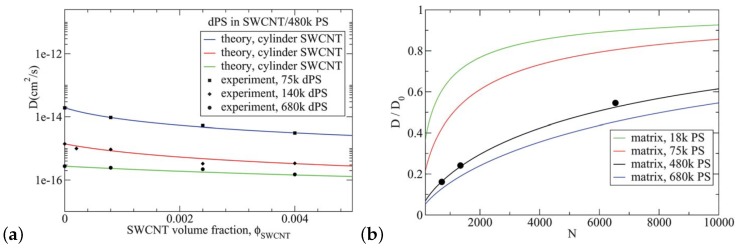
(**a**) Diffusivity of tracer of different molecular weights in an entangled matrix (480 kDa PS) as a function of the SWCNT volume fraction [214] in a PS/SWCNT nanocomposite system: (i) theoretical prediction for 75 kDa dPS (blue line), (ii) 140 kDa dPS (red line), (iii) 680 kDa dPS (green line line) (iv) experimental results [69] for 75 kDa dPS (squares), (v) experimental results for 140 kDa dPS (diamonds), and (vi) experimental results for 680 kDa dPS (circles). (**b**) Normalized diffusivity of tracer in an entangled matrix as a function of the number of monomers of the entangled polymer chains at the SWCNT percolation threshold ($\phi_{\mathrm{SWCNT}}=0.004$): (i) theoretical prediction for 18 kDa PS matrix (green line) (ii) 75 kDa PS matrix (red line) (iii) 480 kDa PS matrix (black line) (iv) 680 kDa PS matrix (blue line) (v) experimental results for PS/SWCNT system in 480 kDa PS matrix at $\phi_{\mathrm{SWCNT}}=0.004$ (symbols) [69]. Reproduced from [214] with permission from the Royal Society of Chemistry.

A theory based on the reptation model was developed for PS/SWCNT nanocomposites, which was modified to incorporate the effect of the surface monomer contacts. In particular, it predicted well the experimental tracer PS diffusion for low SWCNT loading below the percolation threshold as can be seen in Figure 16. In addition, the theory could predict the effects of matrix molecular weight (in agreement with experiments), nanoparticles geometry and diameter of SWCNT on the PS diffusion [214].

**Figure 17:**
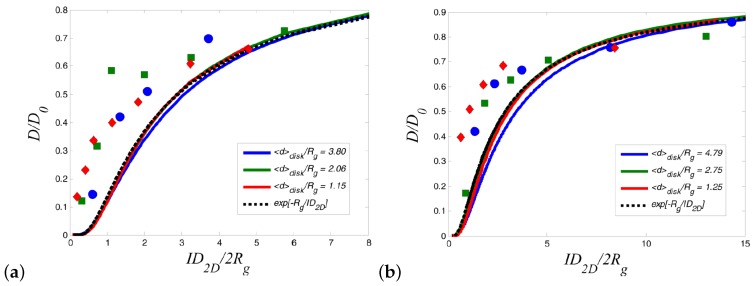
(**a**) Plot of reduced diffusion coefficient [215] versus the confinement parameter for the poly(styrene) data from Martin et al. [216]. (**b**) Reduced diffusion coefficient versus 2D confinement parameter $I\;D_{2D}/2R_{g}$ for the deuterated PMMA/PMMA couple from Lin et al. [217]. Reproduced from [215] with permission from AIP.

An excluded volume analytical model predicts the decrease of polymer diffusion in nanocomposites, in agreement to experiments at low nanoparticle loading (lower $I\;D/2R_{g}$ values) [215]. However, at higher loading (low $I\;D/2R_{g}$ values), the model predicts a slower polymer diffusion than that observed in experiments as can be seen in Figure 17. Polymer disentanglement under high loading [15] cannot be accounted for in that analytical model, thus suggesting unknown physical mechanisms are active in that regime.

## 7. Conclusions

We discuss the very challenging problem of reptational diffusion in melts and nanocomposites that has been studied in the last three decades. While CG models such as the Kremer-Grest model can provide us with an understanding of different dynamical regimes and agree with the predictions of the tube reptation model in polymer melts, they lack detailed chemical information. The semiflexibility can be inserted to the CG model, through a bending and a torsion potential, and has an effect on the entangled polymer diffusion and diffusion coefficient dependence on chain length. Detailed chemical information can be incorporated in fully atomistic models; however, these are limited to short chains (oligomers) due to their computational demand thus cannot capture the entangled polymer dynamics. There are different approaches to insert the chemical information, such as united atom models or the IBI method, which have been proved successful in entangled polymer dynamics modeling and diffusion coefficient dependence on chain length for mildly entangled polymer melts (such as PEO, PE, PS, PB). However, these developed models still remain computationally demanding to be applied for the diffusion in polymer nanocomposites. In particular, only the effect of graphite walls on the diffusion of mildly entangled PE chains (using a united atom force field) has been implemented. In addition, the coarse graining by the IBI method is developed at one temperature and an energy renormalization approach [106,107] should be applied to predict the polymer diffusion in different temperatures. However, this method has not still been implemented in nanocomposites.

In future, it is expected that atomistic simulations combined with models of soft potentials (the nonbonded monomers interact through a linear finite force rather than a hard core LJ force), multiblobs [218,219] or slip-springs (since well entangled polymers can be modeled in that method) will be implemented to study reptational polymer diffusion and can be useful in polymer nanocomposites, where diffusion is altered due to nanoparticle fillers size, loading, geometry and polymer-nanoparticles interaction. In general, DPD simulations are faster and can reach a larger length scale than the Kremer-Grest MD simulations. The soft LJ force is independent of the DPD thermostat, and a time step for integration between 0.01–0.02 ps can be applied [162,167] in polymer melts by still conserving the energy and momentum [162] of the system and avoid bond crossings. The slip-spring model combined with DPD simulations can improve much further the speed of polymer melt simulations, up to 500 times faster, using a time step for integration of 0.06 ps [110,191] requiring also smaller number of particles, in comparison to the Kremer-Grest model (time step 0.01 ps). In the implementation of BD combined with slip-springs, in order to model entangled polymer dynamics, a time step for integration of 0.1 ps is used [205].
